# From Clinical Trials to Real-World Practice: Surgical Feasibility and Postoperative Outcomes After Neoadjuvant Chemoimmunotherapy for Locally Advanced NSCLC in a Single-Center Experience

**DOI:** 10.3390/cancers18121914

**Published:** 2026-06-12

**Authors:** Filippo Lococo, Dania Nachira, Khrystyna Kuzmych, Carolina Sassorossi, Chiara Scognamiglio, Leonardo Petracca Ciavarella, Maria Letizia Vita, Virginia Proietti, Alessio Stefani, Elisa Meacci, Guru Tudimella, Maria Teresa Congedo, Alessandra Cancellieri, Emanuele Vita, Emilio Bria, Stefano Margaritora

**Affiliations:** 1 Thoracic Surgery Unit, Catholic University of the Sacred Heart, 00168 Rome, Italy; filippo.lococo@policlinicogemelli.it (F.L.); dania.nachira@policlinicogemelli.it (D.N.); elisa.meacci@unicatt.it (E.M.); emilio.bria@policlinicogemelli.it (E.B.); stefano.margaritora@policlinicogemelli.it (S.M.); 2 Thoracic Surgery Unit, Fondazione Policlinico Universitario A. Gemelli IRCCS, 000168 Rome, Italy; kkristina.kuz@gmail.com (K.K.); kiasco1998@gmail.com (C.S.); leonardo.petraccaciavarella@policlinicogemelli.it (L.P.C.); marialetizia.vita@policlinicogemelli.it (M.L.V.); virgiproietti@gmail.com (V.P.); gurutudimella@gmail.com (G.T.); mariateresa.congedo@policlinicogemelli.it (M.T.C.);; 3 Medical Oncology, Fondazione Policlinico Universitario A. Gemelli IRCCS, 00168 Rome, Italy; alessio.stefani@unicatt.it (A.S.); emanuele.vita@policlinicogemelli.it (E.V.); 4 Pathology Unit, Fondazione Policlinico Universitario A. Gemelli IRCCS, 00168 Rome, Italy; alessandra.cancellieri@policlinicogemelli.it; 5 Medical Oncology Unit, Ospedale Isola Tiberina-Gemelli Isola, 00168 Rome, Italy

**Keywords:** non-small cell lung cancer, neoadjuvant chemo-immunotherapy, pulmonary resection, minimally invasive surgery, pathological complete response, surgical outcomes

## Abstract

Background/Objectives: Perioperative chemo-immunotherapy (CHT-IO) has emerged as a standard treatment strategy for resectable stage II–IIIB NSCLC. However, data regarding surgical feasibility, mini-invasive surgery rates, perioperative outcomes, and postoperative complications in real-world single-center experiences remain limited. Methods: A retrospective single-center analysis was performed including consecutive patients with locally advanced NSCLC treated with perioperative chemo-immunotherapy between March 2024 and March 2026. Patients received platinum-based chemotherapy combined with pembrolizumab or durvalumab, followed by surgical resection with curative intent. Surgical, pathological, and postoperative outcomes were analyzed. Results: Thirty patients received neoadjuvant CHT-IO, of which 25 (83.3%) underwent surgical resection. Reasons for failure to proceed to surgery included treatment-related toxicity or deterioration in performance status (n = 3), disease progression (n = 1), and patient refusal (n = 1). Lobectomy was the most performed procedure (64%), while a minimally invasive approach (uniportal VATS) was adopted in 44% of cases. Moderate-to-severe pleural adhesions (64%) and hilar fibrosis (60%) were observed intraoperatively. Despite these technical challenges, conversion to thoracotomy was required in only one case (4%), no intraoperative complications occurred, and complete (R0) resection was achieved in 96% of patients. Pathological complete response and major pathological response were observed in 36% and 52% of cases, respectively. Postoperative complications occurred in 56% of patients, although most were Clavien–Dindo grade I–II. The presence of comorbidities was the only factor associated with an increased risk of postoperative complications (OR 10.00, 95% CI 0.99–100.46; *p* = 0.05). Median length of hospital stay was 5.65 ± 2.04 days. One postoperative death due to septic complications was recorded. Conclusions: In this real-world monocentric experience, the combination of perioperative CH-ICIs and surgical resection (including mini-invasive approach) was safe and feasible in patients with locally advanced NSCLC. High rates of complete (R0) resection and encouraging pathological responses were observed, consistent with outcomes reported in randomized trials. Although surgery was overall frequently technically demanding, these changes did not appear to compromise perioperative safety or oncological radicality, even when minimally invasive approaches were adopted. Larger studies with longer follow-up are needed to better define long-term oncological outcomes.

## Simple Summary

Patients with locally advanced non-small cell lung cancer are increasingly treated with a combination of chemotherapy and immunotherapy before surgery. Although this strategy has shown promising results in clinical trials, concerns remain regarding the mini-invasive surgical approach and its feasibility and safety in everyday clinical practice, particularly because treatment-related inflammation and fibrosis may increase surgical complexity. In this real-world single-center study, we evaluated the surgical and pathological outcomes of patients treated with preoperative chemo-immunotherapy followed by lung resection. Our findings showed high rates of complete tumor resection and encouraging pathological responses, despite technically demanding operations related to treatment-induced tissue changes. Minimally invasive surgery was feasible in selected patients with a low conversion rate, and no intraoperative complications were observed in the overall cohort. Overall, these results support that surgery (even mini-invasive surgery) after chemo-immunotherapy can be safe and feasible for locally advanced lung cancer and provide further real-world insight into the surgical management of these patients.

## 1. Introduction

Non-small cell lung cancer (NSCLC) represents the most prevalent form of lung cancer. While surgery remains the cornerstone of treatment for early-stage tumors, optimal management of locally advanced NSCLC is more complex and relies on the integration of locoregional therapies, including surgery and radiotherapy, with systemic treatment strategies. In this context, the therapeutic landscape of locally advanced NSCLC has been substantially reshaped by the integration of immune checkpoint inhibitors (ICIs). Following the initial success in the metastatic setting, where durable responses and improved overall survival were demonstrated, several randomized controlled trials (RCTs) subsequently showed a clear benefit of neoadjuvant and perioperative chemo-immunotherapy in patients with locally advanced EGFR/ALK wild type NSCLC [1,2,3,4]. Collectively, these trials demonstrated significant improvements in event-free survival (EFS), overall survival (OS) and pathological complete response (pCR) rates compared with chemotherapy alone.

Administering ICIs while the primary tumor is in situ may provide several theoretical advantages, including a greater antigen exposure, potentially facilitating more robust tumor recognition and promoting the early development of systemic immune memory.

In recent years, several studies have reported initial real-world experiences from different countries, most of them based on multicentric data collection [5,6,7]. Evaluating outcomes in a real-world patient population is particularly important as clinical trials often adopt rigorous enrollment criteria, preferentially selecting younger and fitter patients. Consequently, it remains essential to determine whether these outcomes are reproducible in “real-world” clinical practice, where patients are often older, present with a greater burden of comorbidities, and may face different clinical and logistical challenges. Furthermore, concerns persist regarding the technical complexity of surgery following induction of CHT-ICI therapy, particularly because of treatment-related hilar inflammation and fibrosis.

However, multicentric data, especially if retrospectively collected, are inevitably affected by inter-institutional heterogeneity, including differences in inclusion criteria, staging methodology, different definitions of resectability and consequently surgical approaches, all of which may represent potential sources of bias.

In this context, the present study aimed to evaluate the feasibility, safety and early outcomes of perioperative chemo-immunotherapy followed by pulmonary resection in a monocentric real-world cohort of patients with locally advanced (LA) NSCLC, integrating our findings with evidence from RCTs and recently published multi-institutional real-world series.

## 2. Materials and Methods

### 2.1. Study Population

The study was approved by the Ethical Committee of the Università Cattolica del Sacro Cuore (Approval Code ID:0000781/2025) and conducted in accordance with the ethical principles of the Declaration of Helsinki and its subsequent amendments. Written informed consent was obtained from all participants prior to inclusion. Data collection and reporting adhered to the Strengthening the Reporting of Observational Studies in Epidemiology (STROBEs) guidelines.

All consecutive patients with stage IIA to IIIB NSCLC, according to the 9th edition of AJCC Cancer Staging Manual, who received (4 cycles) perioperative platinum-based chemotherapy in combination with pembrolizumab (n = 21) or durvalumab (n = 9) were retrospectively analyzed. Specifically, the study included both patients treated with durvalumab (from March 2024) and patients receiving pembrolizumab (from March 2025 onward), following its approval and reimbursement in Italy.

According to institutional policy, clinical, pathological and surgical data were prospectively collected and retrospectively analyzed.

### 2.2. Inclusion/Exclusion Criteria and Staging/Restaging Procedures

The present study is a retrospective analysis of selected patients with LA-NSCLC treated with perioperative chemo-immunotherapy followed by pulmonary resection. Accordingly, not all consecutive patients with stage II–III disease received this treatment strategy during the study period, as treatment allocation depended on multiple clinical and multidisciplinary factors.

#### 2.2.1. Inclusion Criteria

- Eastern Cooperative Oncology Group (ECOG) performance status 0–2;
- Patients with LA-NSCLC (stage IIA to IIIB-N2 only) considered “resectable” following multidisciplinary tumor board evaluation and EORTC criteria [8];
- Absence of epidermal growth factor receptor (EGFR) mutations or anaplastic lymphoma kinase (ALK) alterations at molecular testing in patients with lung adenocarcinoma.

#### 2.2.2. Exclusion Criteria

- Patients who underwent surgery (“salvage surgery”) after CHT-ICI treatment performed in other settings;
- Patients enrolled in clinical trials;
- Patients with other primary lung cancer;
- Patients with contraindications to perform immunotherapy;
- Presence of EGFR/ALK mutations/alterations at molecular testing in patients with lung adenocarcinoma;
- Insufficient tumor tissue to perform molecular profiling in patients with diagnosis of lung adenocarcinoma.

All patients underwent a standard pre-treatment staging workup, including tumor biopsy, contrast-enhanced total body computed tomography (CT), positron emission tomography/CT scan (PET-CT), and invasive mediastinal nodal staging with endobronchial ultrasound (EBUS) or endoscopic ultrasonography (EUS), as indicated. Brain magnetic resonance imaging (MRI) was also performed, when clinically indicated.

Following completion of ICI treatment, all patients underwent restaging with contrast-enhanced total body CT scan before surgical resection, while PET-CT scan and EBUS/EUS re-biopsy were not routinely performed in this setting. In detail, PET CT scan was performed only in doubtful cases to confirm extra-thoracic disease and endoscopic re-biopsy of lymph nodal disease only when a pseudo-progression was suspected.

### 2.3. Surgical Techniques

Surgery was scheduled within 6 weeks after completion of the last cycle, in accordance with timelines adopted in major RCTs [1,2,3,4], although delays could occur due to clinical or logistical factors inherent to routine practice.

It was planned for all patients to undergo anatomic pulmonary resection with systematic lymph node dissection according to institutional standards and current international guidelines [9,10].

Surgery was performed by qualified and experienced thoracic surgeons with expertise in complex thoracic surgery procedures. The choice between lateral thoracotomy or minimally invasive approach was based on tumor location, size, presence of hilar involvement, and surgeon’s judgment and experience. Whenever a minimally invasive approach was considered, uniportal video-assisted thoracoscopic surgery (U-VATS) represented the standard approach adopted at our institution.

Surgical data including operative approach, extent of resection, operative time, intraoperative technical findings and complications, postoperative morbidity, hospital length of stay, and other details related to the operative experience were prospectively collected. The intra- and postoperative findings and complications were defined and standardized as in clinical practice.

### 2.4. Pathological Examination

Pathological examination was performed by specialized thoracic pathologists (blinded to clinical information and treatment allocation) according to the pan-tumor immune-related pathological response criteria by the International Association for the Study of Lung Cancer and the tumor regression grading system by Junker [11,12].

Similarly, pathological complete response (pCR) was defined as the absence of residual viable tumor cells in the primary tumor and sampled lymph nodes, whereas major pathological response (MPR) was defined as the presence of less than 10% of residual viable tumor cells in the primary tumor and sampled lymph nodes, including cases achieving pCR.

### 2.5. Outcomes

The primary outcome of the study was to evaluate the feasibility of the multimodal treatment (peri-operative CHT-ICIs and surgical resection) in terms of attrition to surgery, intraoperative surgical outcomes and postoperative complications.

Secondary outcomes were to evaluate potential factors associated with postoperative complications. Oncological follow-up is ongoing and the mean follow-up was 26.92 ± 23.90 months (median = 14) at the time of completing this manuscript.

### 2.6. Statistical Analysis

Continuous variables are presented as mean ± standard deviation, whereas categorical variables are reported as absolute frequencies and percentages (%). The Kolmogorov–Smirnov test was used to assess the normality of data distribution. Categorical variables were compared using the Chi-square test. Continuous variables were analyzed with the independent sample Student’s t-test when normally distributed, or with the Mann–Whitney U test when normality was not met.

Univariable regression analysis was performed to identify the main risk factors associated with postoperative complications. Due to the exploratory nature of the study, the limited sample size and the low events-per-variable ratio, a forward stepwise logistic regression approach was adopted to screen potential factors associated with postoperative complications while limiting the number of covariates retained in the final model. Results should therefore be interpreted as exploratory and hypothesis-generating. Only clinically relevant variables with a *p*-value < 0.20 in the univariable analysis were included in the multivariable regression model.

A *p*-value < 0.05 was considered statistically significant. Statistical analyses were performed using IBM SPSS Statistics for Macintosh (version 25.0; IBM Corp., Armonk, NY, USA).

## 3. Results

As reported in the Consort Diagram, between March 2024 and March 2026, among the 30 patients who started CHT-ICI treatment, 25 (83%) underwent surgical resection with curative intent. Reasons for not receiving surgery were treatment-related adverse events (Hematologic TRAEs, one pts) or deterioration in performance status (two patients), cancer progression (one pt) and patient refusal (one pt candidate to left pneumonectomy) with a total proceeding rate to surgery of 83.3%.

The clinical data from 25 patients, who underwent neoadjuvant chemo-immunotherapy followed by surgical treatment for a clinical stage IIb–IIIb NSCLC at our center, were reviewed. Fifteen patients (60%) were male; five patients (20%) were current smokers, while 19 (76%) were former smokers. The other main clinicopathological characteristics of the cohort are summarized in Table 1.

In 18 cases (72%), the histology was adenocarcinoma, with a PDL1 ≥ 50% in 44% of cases.

As reported in Table 1, the majority of patients had stage III at diagnosis (92%) and N2-disease (60%).

Seven patients (28%) experienced chemo-related grade ≤ 2 TRAEs that were all clinically manageable with no interruption of scheduled protocols. Among surgically treated patients, nine (36%) experienced a delay of more than 6 weeks from the last CHT-IOT pulse due to logistic factors (n = 7) or medically manageable toxicities (n = 2).

The main type of resection was lobectomy (16 cases, 64%), followed by pneumonectomy in four cases (16%), while an exploratory thoracotomy was never performed (see Table 2). The mean operative time was 179.64 ± 39.54 min. Intraoperatively, moderate-to-severe pleural adhesions were observed in 64% of cases, while moderate-to-severe fibrosis in 60%. A minimally invasive approach, particularly U-VATS, was adopted in 11 patients (44%), with an increase over time from 33% in the first 12 months of the study to 54% in the subsequent period (13–24 months).

Conversion to thoracotomy was required only in one case (4%) due to technical difficulty, related to inflammation and/or dense adhesions involving the fissure and surrounding hilar and mediastinal nodal stations. Despite these technical challenges, no major intraoperative complications were recorded.

Complete resection (R0) was achieved in 24 patients (96%), and pCR was observed in nine cases (36%). Mean PDL1% was 64.38 ± 29.33 in pCR vs. 33.83 ± 31.45 in not pCR cases (*p* = 0.042). Otherwise, when exploring this association considering PD-L1 as categorical variable, the difference was not statistically significant.

The mean length of hospital stay was 5.65 ± 2.04 days (5.25 ± 2.61 after UVATS and 5.81 ± 1.93 after open surgery). Four patients were discharged with a chest tube connected to a Heimlich valve due to prolonged air leak (three after open lobectomy and one after UVATS lobectomy). Only one postoperative death (4%) occurred due to septic complications after an open left upper lobectomy.

Adjuvant RT was performed in a patient with R2-disease with no complication while 17 patients (68%) underwent adjuvant immunotherapy (10/16, 62% of pembrolizumab group and 7/9, 78% of durvalumab group).

Fourteen patients (56%) experienced postoperative complications, mainly grade I–II (40%), according to Clavien–Dindo classification (see Table 2).

When exploring factors associated with postoperative complications, the presence of comorbidities was the only variable independently associated at multivariable analysis (OR, 95%CI: 10.00 [0.99–100.46], and *p* = 0.05), as reported in Table 3.

## 4. Discussion

Although several studies have already reported real-world data on the feasibility and short-term outcomes of a surgical approach after chemo-immunotherapy in locally advanced NSCLC, the present study explores this multimodal treatment in a single-center setting of a high-volume hospital, offering a comprehensive overview for similar centers that are starting this treatment. Our detailed analysis has shown encouraging results regarding the feasibility and outcomes of this multimodal strategy, supporting its implementation in routine clinical practice.

Following the approval of ICIs in combination with platinum-based chemotherapy as neoadjuvant or perioperative treatment for resectable locally advanced NSCLC, based on the positive results of landmark phase III trials such as AEGEAN, CheckMate 816, CheckMate 77T, KEYNOTE-617 [1,2,3,4], there is a growing need to confirm the reproducibility of these outcomes in broader real-world populations.

However, multicentric datasets are often affected by inter-institutional heterogeneity in patient selection, staging procedures, and surgical strategies, as well as variability in definition of “resectability”, all of which may introduce significant confounding factors. By contrast, our study reflects a consistent multidisciplinary decision-making process and a standardized surgical approach, allowing a more controlled evaluation of the true impact of chemo-immunotherapy on surgical feasibility and pathologic outcomes.

Our findings confirm the feasibility of a multimodal treatment strategy with favorable surgical outcomes and postoperative complications. Early-phase evidence, including the study by Bott et al. [13], first established the perioperative safety of neoadjuvant PD-1 blockade, showing that neoadjuvant nivolumab monotherapy was not associated with unexpected attrition to surgery nor increased perioperative mortality or morbidity, with 20 of 22 patients (91%) successfully undergoing resection and no operative mortality. These results were subsequently validated at scale in the CheckMate 816 trial [1], where the addition of nivolumab to neoadjuvant chemotherapy did not compromise surgical feasibility or increased surgery-related adverse events.

Since NSCLC patients with EGFR mutations and ALK rearrangements are generally associated with lower responsiveness to immune checkpoint inhibitors, molecular profiling is therefore essential for appropriate patient selection and therapeutic decision-making, because targeted perioperative strategies may be more beneficial than immunotherapy in these subgroups.

In our cohort, 83.3% of patients (25 of 30) proceeded to surgical resection with curative intent following CHT-ICI treatment, a rate closely aligned with those reported in pivotal trials, such as CheckMate 816 (83% in the nivolumab–chemotherapy arm versus 75% in the chemotherapy-alone arm) [1], thereby supporting the external validity of randomized evidence.

However, real-world studies have demonstrated a wider variability in resection rates [14,15,16]. For instance, Brunelli et al. in a multicentric European cohort [5] reported a remarkably high proceeding-to-surgery rate of 93.2%, whereas Cooper et al. [17] observed a lower resection rate of 78% in a US-based population. This variability likely reflects variations in multidisciplinary decision-making and thresholds for surgical candidacy in locally advanced disease across institutions. In addition, logistical factors and treatment-related toxicity may contribute to delays to surgery and, consequently, higher attrition rates in less controlled settings. Taken together, these findings suggest that outcomes following neoadjuvant chemo-immunotherapy are not solely treatment-dependent but are also influenced by organizational and decision-making processes within multidisciplinary care pathways.

The primary reasons for failure to proceed to surgery in our series were disease progression (n = 1) and treatment-related toxicity or deterioration in performance status (n = 3), consistent with rates reported in real-world series and randomized trials, ranging from 3.8 to 9% and 2.3 to 13%, respectively [1,2,3,4,5,14,15,16,17,18]. Similarly, a recent review estimated that 15–24% of enrolled patients never reach the operating room, most commonly due to radiographic progression (5–8%) or evolving surgical ineligibility [19]. In this context, Cooper et al. identified patient-related factors such as advanced age (>72 years) as a significant predictor of failure to proceed to surgery, likely due to increased susceptibility to treatment-related toxicity and a higher burden of comorbidities [17].

From a surgical standpoint, 11 patients (44%) in our cohort underwent resection via a minimally invasive approach, specifically U-VATS. This rate was higher than the 29.5% reported in the CheckMate 816 trial [1], reflecting the progressive adoption of minimally invasive surgery (MIS) together with increasing surgical expertise, following its publication. However, compared with other real-world series such as that by Brunelli et al. [5], our rate of MIS utilization was lower (44% vs. 66%), while the conversion rate was markedly reduced (4% vs. 19%), suggesting different surgical strategies. In our practice, cases with anticipated technical complexity, such as centrally located tumors, were preferentially managed via upfront thoracotomy, thereby reserving MIS for more selected patients and minimizing the need for intraoperative conversion (only 4% converted to open surgery). Conversely, Brunelli and colleagues, by adopting a broader application of MIS, were exposed to higher conversion rates. Basically, by excluding those cases converted to open surgery, MIS was performed in similar percentages in the two cohorts (40% vs. 47%).

Notably, our surgical strategy has evolved throughout the study period. As the surgical team gained experience in the post-immunotherapy setting, the rate of minimally invasive procedures increased from 33% in the first 12 months of the study to 54% in the second period, reflecting a progressive learning curve and greater surgical confidence with these procedures as they became more routine. Importantly, this evolution mirrors the broader shift towards increased MIS adoption after neoadjuvant chemo-immunotherapy, with meta-analytic data showing MIS approaches in approximately 47% of resections across prospective trials [19].

Despite the low conversion rate, minimally invasive resections were frequently technically demanding. Intraoperative findings commonly included dense vascularised adhesions, hilar inflammation and treatment-related fibrosis, with moderate-to-severe pleural adhesions observed in a substantial proportion of cases and hilar fibrosis documented in 60% of patients. Furthermore, the relatively low number of retrieved lymph nodes may indirectly reflect the increased technical complexity encountered during systematic nodal dissection in this context. Nevertheless, these complexities did not translate into adverse intraoperative outcomes. No intraoperative complications were recorded, and complete (R0) resection was achieved in 96% of patients. These findings support the concept that, although surgery after chemo-immunotherapy requires substantial technical adaptation, treatment-induced tissue remodeling does not necessarily impair oncological radicality or perioperative safety when procedures are performed in experienced centers.

The rate of pneumonectomies in our series was approximately 15% (all left side), aligning with that reported in major RCTs [1,2,3,4,10]. By contrast, Brunelli et al. [5] reported a notably lower pneumonectomy rate (only 4.1%) in their multicenter cohort, which is substantially lower than the rates reported in the aforementioned trials. This discrepancy may be partly explained by differences in patient selection. As acknowledged by the authors, patients with stage III NSCLC and borderline resectability may have been more frequently directed towards non-surgical management, such as the PACIFIC regimen [20], thereby reducing the proportion of patients undergoing pneumonectomy. In our experience, we did not exclude those cases where pneumonectomy was planned at the beginning; this also explains a higher pneumonectomy rate compared with some clinical trial (i.e., AEGEAN [2]) where these cases were excluded from enrolment.

While about half of population experienced postoperative complications, these were mainly grade I–II (40%) according to Clavien–Dindo classification, suggesting an acceptable toxicity profile after surgery. Regarding the 90-day mortality, one postoperative death was observed in our cohort, corresponding to an overall mortality rate of 4%. Although not negligible, this figure remains within the range (from 2.5% to 5.8%) reported in contemporary real-world series [5,14,15,16].

From an oncological perspective, our series demonstrated a relatively high pathological complete response (nine/25 patients, 36%) and MPR (13/25 patients, 52%), with no pathological response (NPR) observed in 16% of cases. In comparable trials and cohorts, MPR rates have generally ranged from 47% to 52.7%, with pCR rates between 30% and 31.4%, remaining consistent across clinical stages [1,2,3,4,5,17]. Despite the high % of PD-L1 detected in our cohort and the association between %PD-L1 with pCR (*p* = 0.042) as similarly reported by other authors [1,14,21,22], which may potentially explain our high rate of pCR/MPR, the limited sample size of this monocentric population may disproportionately influence percentage-based outcomes, and these findings should therefore be interpreted accordingly.

Taken together, our findings, in line with two recent meta-analyses [23,24] and the more recent trials (Neotorch [25], NADIM [26], RATIONALE-315 [27], TD-FOREKNOW [28] and NEOpredict-Lung [29]), support the reproducibility of chemo-immunotherapy outcomes in real-world clinical practice. More importantly, our data suggest that the integration of neoadjuvant immunotherapy into multimodal treatment pathways may be achieved without compromising surgical feasibility, oncological radicality, or perioperative safety in resectable locally advanced NSCLC.

### Limitations

This study is subject to several limitations inherent to its retrospective design. First, potential bias in the collection and definition of variables cannot be excluded. Second, selection bias should be considered when interpreting these results, as patients who did not receive neoadjuvant therapy during the same period, potentially due to comorbidities, frailty, or both, were excluded and may have had a poorer prognosis. Indeed, as reported above, not all consecutive patients with stage II–III disease received this treatment strategy during the study period, as treatment allocation depended on multiple clinical and multidisciplinary factors. Moreover, we do not exclude other cases with similar characteristics undergoing different treatments at another location after a multi-disciplinary discussion following the selection criteria reported above.

Concerning statistical analysis, the use of a forward conditional logistic regression approach, which is commonly used in preliminary clinical analyses, may have introduced model instability and variable-selection bias, particularly given the small sample size. Indeed, the moderate predictive performance and relatively low explained variance indicate that other unmeasured clinical factors are likely involved in the development of postoperative complications. Therefore, the identified associated factors to postoperative complications should be interpreted cautiously and validated in larger prospective cohorts.

Finally, the present analysis is limited to surgical and pathological outcomes. Given the relatively short follow-up period, survival data are not yet mature and will be addressed in future analyses.

## 5. Conclusions

In this single-center real-world cohort, neoadjuvant chemo-immunotherapy followed by surgical resection was feasible and safe in patients with locally advanced NSCLC, achieving high rates of complete resection and encouraging pathological responses, consistent with evidence from randomized trials. Although factors such as the limited sample size and the higher proportion of patients with elevated PD-L1 expression may influence the interpretation of our findings, the observed outcomes remain broadly consistent with those reported in clinical trials and real-world studies. These findings support the applicability of this multimodal approach in routine clinical practice, despite the heterogeneity of real-world populations. The key elements for a successful multimodal protocol within the immunotherapy era are the need for multidisciplinary coordination, experienced thoracic surgeons, and treatment centralization in resectable locally advanced NSCLC.

Further studies with larger cohorts and longer follow-up are needed to confirm the impact of this strategy on long-term oncological outcomes and to better define patient selection.

## Figures and Tables

**Table 1:** Clinicopathological characteristics of the whole population.

Variable	Patients (n = 25)
Sex (male)	15 (60%)
Age (years)	67.55 ± 7.92
Smoking status	
Never	1 (4%)
Active	5(20%)
Former smoker	19 (76%)
BMI, kg/m^2^	24.68 ± 2.32
Comorbidity	17 (68%)
Comorbidity type	
Cardiovascular	9 (36%)
COPD	9 (36%)
Chronic kidney disease	2 (8%)
Diabetes mellitus type II	4 (16%)
Previous malignancy	6 (24%)
cStage (9th edition TNM)	
IIA	1 (4%)
IIB	1 (4%)
IIIA	18 (72%)
IIIB (n2-only)	5 (20%)
cT-status	
T1	2 (8%)
T2	5 (20%)
T3	11 (44%)
T4	7 (28%)
cN-status	
N0	1 (4%)
N1	9 (36%)
N2a	11 (44%)
N2b	4 (16%)
PDL1 < 1%	5 (20%)
1% ≤ PDL1 < 50%	9 (36%)
PDL1 ≥ 50%	11 (44%)
Immunotherapy drug	
Pembrolizumab	16 (64%)
Durvalumab	9 (36%)
(CHT) Treatment-related adverse effects	7 (28%)
Complication grade (CTCAE classification)	
Grade 1	3 (12%
Grade 2	3 (12%)
Grade 3	1 (4%)
Grade 4	0
Grade 5	0
ycStage	
0	8 (32%)
IA2	2 (8%)
IA3	2 (8%)
IB	3 (12%)
IIA	4 (16%)
IIB	2 (8%)
IIIA	4 (16%)

Footnote: Data are presented as mean ± standard deviation or n (%), as appropriate. BMI: body mass index; COPD: chronic obstructive pulmonary disease; CTCAE: Common Terminology Criteria for Adverse Events; PDL1: programmed death-ligand 1.

**Table 2:** Surgical, pathological, and postoperative outcomes of the study population.

Variable	Patients (n = 25)
Surgical approach	
Thoracotomy	14 (56.0%)
U-VATS	11 (44.0%)
Operative time (minutes)	179.64 ± 39.54
Conversion	1 (4%)
Type of surgery	
Pneumonectomy (left = 4; right = 0)	4 (16%)
Bilobectomy	3 (12%)
Lobectomy	16 (64%)
Segmentectomy	2 (8%)
Side of surgery (right)	14 (56%)
Pleural adhesions	
Absent	9 (36%)
Moderate	13 (52%)
Severe	3 (12%)
Hilar fibrosis	
Absent	10 (40%)
Moderate	7 (28%)
Severe	8 (32%)
Intraoperative complications	0
ICU admission	7 (28%)
Histology	
Fibrosis	1 (4%)
Adenocarcinoma	18 (72%)
Squamous cell carcinoma	6 (24%)
N1 nodes removed	6.88 ± 5.17
N2 nodes removed	4.60 ± 1.26
Total number of nodes removed	14.60 ± 7.67
R status	
R0	24 (96%)
R1-R2	1 (4%)
ypStage	
0	9 (36%)
IA1	2 (8%)
IA2	2 (8%)
IIA	1 (4%)
IIB	3 (12%)
IIIA	6 (24%)
IIIB	2 (8%)
Response grade (Junker classification)	
I	3 (12%)
IIA	10 (40%)
IIB	2 (8%)
III	10 (40%)
Major pathological response (MPR)	13 (52%)
-Complete pathological response (CPR)	9 (36%)
Partial pathological response (PPR)	8 (32%)
No pathological response (NPR)	4 (16%)
Chest tube duration (days)	6.04 ± 2.94
Postoperative complications	14 (56%)
Clavien–Dindo Classification	
0	11(44%)
I	3 (12%)
II	6 (24%)
III	4 (16%)
IV	1 (4%)
Adjuvant therapy	17/25 (68%)
30-day mortality	1 (4%)
90-day mortality	1 (4%)
Hospital stay (days)	5.65 ± 2.04
Discharge with Heimlich valve for prolonged air leak	4 (16%)

Footnote: Data are presented as mean ± standard deviation or n (%), as appropriate. CPR: complete pathological response; ICU: intensive care unit; MPR: major pathological response; NPR: no pathological response; R status: residual tumor classification; U-VATS: uniportal video-assisted thoracoscopic surgery; ypStage: post-treatment pathological stage.

**Table 3:** Univariable and multivariable analyses of risk factors associated with postoperative complications.

	Univariable Analysis		Multivariable Analysis	
	OR [95% CI]	*p*-Value	OR [95% CI]	*p*-Value
Age	1.05 [0.95–1.17]	0.376		
BMI	0.88 [0.61–1.25]	0.484		
ASA score	0.28 [0.07–2.03]	0.268		
Comorbidity	0.10 [0.02–1.01]	0.03	10.00 [0.99–100.46]	0.05
Smoke	0.95 [0.22–4.25]	0.514		
<1% PDL-1 > 50%	0.23 [0.04–1.30]	0.087		
Hilar fibrosis	0.82 [0.32–2.09]	0.152		
Type of surgical resection	0.47 [0.15–1.42]	0.352		
Pleural adhesions	1.15 [0.34–3.89]	0.111		
Number of lymph nodes removed	1.04 [0.94–1.16]	0.472		

## Data Availability

The datasets generated and/or analyzed during the current study are available from the corresponding author on reasonable request.

## Abbreviations

The following abbreviations are used in this manuscript:

AJCC	American Joint Committee on Cancer
ALK	Anaplastic lymphoma kinase
BMI	Body mass index
CHT-ICI	Chemo-immunotherapy
CI	Confidence interval
COPD	Chronic obstructive pulmonary disease
CT	Computed tomography
CTCAEs	Common Terminology Criteria for Adverse Events
DFS	Disease-free survival
EAP	Expanded access program
EBUS	Endobronchial ultrasound
ECOG	Eastern Cooperative Oncology Group
EFS	Event-free survival
EGFR	Epidermal growth factor receptor
EUS	Endoscopic ultrasonography
IASLC	International Association for the Study of Lung Cancer
ICI	Immune checkpoint inhibitor
ICU	Intensive care unit
LA-NSCLC	Locally advanced non-small cell lung cancer
MIS	Minimally invasive surgery
MPR	Major pathological response
MRI	Magnetic resonance imaging
NSCLC	Non-small cell lung cancer
NPR	No pathological response
OR	Odds ratio
OS	Overall survival
pCR	Pathological complete response
PD-L1	Programmed death-ligand 1
PET-CT	Positron emission tomography/computed tomography
PPR	Partial pathological response
RCT	Randomized controlled trial
STROBE	Strengthening the Reporting of Observational Studies in Epidemiology
TNM	Tumor, node, metastasis
U-VATS	Uniportal video-assisted thoracoscopic surgery
